# Benefits of Gardening Activities for Cognitive Function According to Measurement of Brain Nerve Growth Factor Levels

**DOI:** 10.3390/ijerph16050760

**Published:** 2019-03-02

**Authors:** Sin-Ae Park, A-Young Lee, Hee-Geun Park, Wang-Lok Lee

**Affiliations:** 1 Department of Environmental Health Science, Sanghuh College of Life Science, Konkuk University, Seoul 05029, Korea; danapre@nate.com; 2 Sport Science Center, Daejeon 35021, Korea; exepre@cnu.ac.kr; 3 Department of Sport Science, Chugnam National University, Daejeon 34134, Korea; leewl@cnu.ac.kr

**Keywords:** complementary and medicine, exercise intervention, horticultural therapy, socio horticulture, older adults

## Abstract

The objective of this study was to determine the effects of gardening activities in senior individuals on brain nerve growth factors related to cognitive function. Forty-one senior individuals (age 76.6 ± 6.0 years) were recruited from the local community in Gwangjin-gu, Seoul, South Korea. A 20-min low-to-moderate intensity gardening activity intervention, making a vegetable garden, was performed by the subjects in a garden plot located on the Konkuk University (Seoul, South Korea) campus. The gardening involved six activities including cleaning a garden plot, digging, fertilizing, raking, planting/transplanting, and watering. To determine the effects of the gardening activities on brain nerve growth factors related to memory, blood samples were drawn twice from each subject before and after the gardening activity by professional nurses. The levels of brain nerve growth factors, including brain-derived neurotrophic factor (BDNF), vascular endothelial growth factor (VEGF) and platelet derived growth factor (PDGF), were analyzed. Levels of BDNF and PDGF were significantly increased after the gardening activity. This study revealed a potential benefit of gardening activities for cognitive function in senior individuals.

## 1. Introduction

Brain function and memory decline in the aging process [1]. The volume and weight of the brain decrease at a rate of approximately 5% per decade after 40 years of age, with the actual rate of decline possibly increasing with older age, particularly > 70 years [2,3]. The hippocampus is a small organ located within the brain’s medial temporal lobe, and is associated primarily with memory [4,5,6]. A large hippocampal size is associated with good memory and cognitive function; however, atrophy of the hippocampus is associated with the development of dementia [7]. In Alzheimer’s disease and other forms of dementia, the hippocampus is one of the first regions of the brain to exhibit damage such as short-term memory loss and disorientation [8,9].

Previous studies have reported that participation in physical activity is associated with increased memory and cognition [10,11]. Acute aerobic physical activity has been shown to improve cognitive function [12,13,14], short-term memory and long-term memory [15,16,17,18,19,20]. Long-term cardiovascular exercise has been associated with improvement in short-term and long-term memory [20,21,22].

Brain nerve growth factors related to memory and cognitive function, such as brain-derived neurotrophic factor (BDNF) and vascular endothelial growth factor (VEGF), are currently considered to be key proteins that are up-regulated after exercise [23,24,25] and can promote cell proliferation and growth, as well as neuronal development and functioning [26,27]. Moreover, platelet-derived growth-factor (PDGF) has been shown to promote blood vessel growth and neuronal survival [28,29]. Exercise has been shown to increase BDNF expression in the hippocampus and 0-cortical regions [25,30,31,32,33], and increases in BDNF levels have been associated with exercise-induced benefits in hippocampal-dependent memory [33].

Previous studies have reported that gardening can be considered aerobic and muscular exercise [34,35,36]. A 12-week gardening intervention improved physical functional ability, the immune system, muscle strength and flexibility, and reduced blood pressure, waist circumference, and cholesterol levels [37,38]. Various common gardening activities, such as digging, raking, planting/transplanting, weeding, watering and harvesting, are considered to be low-to-moderate intensity physical activities in seniors [35,39,40]. Moreover, the benefits of cognitive function and memory resulting from gardening intervention as a physical activity have been described. For example, a 20-session horticultural therapy program, designed with common gardening activities, significantly improved cognitive function in senior individuals with dementia [41]. Another study reported improved cognitive function in senior individuals with dementia who participated in an 18-session horticultural therapy program compared with a control group [42].

However, there is insufficient research examining the effects of gardening as a physical activity on cognitive function according to measurement of brain nerve growth factor levels. Accordingly, the objective of this study was to measure changes in key proteins related to cognitive function, such as BDNF, PDGF, and VEGF, through short-term, low-to-moderate intensity gardening activity in elderly individuals > 65 years of age.

## 2. Materials and Methods

### 2.1. Subjects

To recruit subjects aged over 65 years, the researchers visited community and welfare centers located in Gwangjin-gu, Seoul, South Korea. After providing an explanation about the study and a flyer, including study objectives, procedure, measurements and schedule, 41 individuals aged over 65 years volunteered to participate in this study. Inclusion criteria were that participants were aged over 65 years, with no physical disability, and no severe chronic disease. Requirements for participation in this study were fasting for 9 h before blood sampling and completing a 20 min gardening activity. Informed written consent was obtained from each subject before starting the study.

A questionnaire, including questions regarding age, sex, current diseases and medications, was completed by the subjects. Before starting the gardening activity, each subject’s body composition, including body weight (g), lean mass (g), fat mass (g), percent fat (%), and body mass index (BMI), were measured using a body fat analyzer (ioi 353; Jawon Medical, Gyeongsan, South Korea). Height was measured using an anthropometer (Ok7979; Samhwa, Seoul, South Korea) without shoes. 

On completion of the study, the subjects received an incentive (approximately $20 USD). This study was approved by the Institutional Review Board of Konkuk University (7001355-201711-HR-212).

A 20-min gardening activity intervention with low-to-moderate intensity gardening activities was designed as a physical activity intervention, with an average of 3.5 metabolic equivalents (METs) based on previous studies (Table 1) [35]. The intensity of physical activity can be expressed as METs [43]. METs > 3 to 6 indicate moderate-intensity physical activity, while METs < 3 represent low intensity [43]. In a previous study, gardening activities, such as digging, raking, and fertilizing, were considered to be moderate-intensity physical activities in individuals aged over 65 years [35]. Gardening activities, such as planting/transplanting and watering using watering cans, were low-intensity physical activities in older adults aged over 65 years. The Centers for Disease Control and Prevention recommend at least 30 min of moderate-intensity physical activity on most days of the week for health maintenance and improvement [43,44].

In the present study, the gardening activity intervention consisted of six activities, including cleaning a garden plot (3.4 METs) for 2 min, digging (4.5 METs) for 5 min, fertilizing (4.0 METs) for 3 min, raking (3.4 METs) for 3 min, transplanting plants (2.9 METs) for 5 min, and watering using a watering can (2.8 METs) for 2 min (Table 1) [35]. The gardening activity was performed in a garden plot previously prepared on the Konkuk University campus. The subjects visited the Konkuk University campus once for this study and were assigned to a garden plot (1 × 1.8 m) for the gardening activity. The subjects were asked to wear comfortable clothes and shoes for gardening. Weather conditions during gardening were temperature 5.6 °C and relative humidity 50.0% (Meteorological Administration of South Korea).

### 2.2. Assessments

To measure changes in brain nerve growth factors that are related to hippocampal blood flow, volume, and memory performance, BDNF, PDGF, and VEGF were analyzed. Three professional nurses obtained a 7 mL blood sample twice from each subject before and after the 20 min gardening activity, respectively. The blood samples were subsequently stored in vacutainers packed in ice and transferred to a laboratory at Chungnam National University (Daejeon, South Korea) for analysis. The blood was centrifuged and the serum was stored in microcentrifuge tubes (Eppendorf, USA) in a freezer at −80 °C. Factors were measured according to protocols supplied by the manufacturer, performed using sandwich ELISA kits for BDNF, PDGF, and VEGF (AbCAM systems, Cambridge, MA, USA). Readings were performed using a microplate reader (Bio-Rad, Hercules, CA, USA) adjusted to a wavelength of 490 nm.

### 2.3. Data Analysis

Demographic information was analyzed using spreadsheet software in Excel (Office 2016; Microsoft Crop., Redmond, WA, USA). To analyze differences in factors, such as BDNF, PDGF, and VEGF, before and after the gardening activity program, a paired *t*-test was performed. To compare gender difference for the BDNF, PDGF, and VEGF, independent samples *t*-test was used; *p* < 0.05 was considered to be statistically significant. Statistical analyses were performed using SPSS (24.0 for windows; IBM Corp., Armonk, NY, USA).

## 3. Results

### 3.1. Demographic Information

The characteristics of the subjects who participated in this study are summarized in Table 2. The mean age of the subjects was 76.6 ± 6.0 years, with males and females comprising 31.7% and 68.3% of the cohort, respectively. The mean BMI of the subjects was 25.4 ± 3.8 kg/m^2^. which is in the overweight range, and 48.8% of the subjects had at least one chronic disease.

### 3.2. Levels of the Brain Nerve Growth Factors

A gardening activity with low-to-moderate intensity, making an vegetable garden, improved levels of the brain nerve growth factors BDNF and PDGF, which are related to memory, in senior individuals who participated in this study (Table 3). There was no significant difference between male and female participants for the brain nervous growth factors (Table 4).

## 4. Discussion

BDNF is a member of the neurotrophin family of factors that supports neural survival, growth and synaptic plasticity, and is highly concentrated in the hippocampus and cortex [45,46,47]. Although decreased levels of these factors have been associated with age-related hippocampal dysfunction and memory impairment, increased BDNF levels resulting from aerobic exercise appears to ameliorate hippocampal deterioration and improve memory function [48,49]. Habitual physical activity is associated with BDNF. In a well-designed randomized controlled trial of 1-year duration of exercise training involving 120 healthy senior individuals, Erickson et al. reported that aerobic exercise increased serum BDNF levels and spatial memory [50]. Another randomized controlled trial also demonstrated an increase in resting BDNF levels after 6 months of aerobic exercise, and cognitive function improved significantly in healthy elderly subjects [51]. In this study, the 20-min gardening activity intervention with low-to-moderate intensity as a physical activity intervention led to increased BDNF concentration in the blood level of the participants. The increase of this key protein, BDNF, by gardening activity intervention can promote memory by increasing cell proliferation and growth, as well as neuronal development and functioning in the hippocampus and cortical regions [25,26,28,30,31,32,33].

Additionally, a 5-week moderate-intensity aerobic training program resulted in a significant increase in resting plasma BDNF concentrations in healthy young men [52]. Serum BDNF concentrations did not significantly change during 10 min of moderate exercise in the warm-up period in healthy male athletes; however, serum BDNF concentrations significantly increased following a ramp test to exhaustion. The increases in BDNF were positively related with exercise-induced changes in anterior hippocampal volume. BDNF is a crucial mediator of exercise-induced neuroplasticity [48]. The beneficial effects of exercise on cognitive function were inhibited when blocking the action of BDNF signaling in the hippocampus [33].

PDGF has been highlighted as a potential new player in neurovascular crosstalk [53]. PDGF has been shown to promote blood vessel growth and neuronal survival [27,29]. In previous studies in the field of exercise, expression of the PDGF gene in human peripheral blood mononuclear cells increased after 30 min of cycling at 80% of peak oxygen uptake in healthy men [54]. Serum levels of PDGF increased significantly from 1700 pg/mL to 4640 pg/mL after an average of 17 min of physical exercise [55]. Accordingly, this study showed similar results to the previous physical exercise studies. The gardening activity intervention as a physical activity could increase PDGF gene levels, promoting vessel growth and neuronal survival [27,29].

In contrast, there was no significant improvement of VEGF levels in this study. VEGF is a hypoxia-inducible protein that promotes the formation and growth of blood vessels, and has also been associated with improved cognition [56,57]. VEGF is expressed in multiple cells and tissues, including smooth and skeletal muscle, endothelial cells, macrophages and glial cells [29,58]. Voss et al. reported that increases in VEGF concentration made the brain more resistant to functional and structural neurodegeneration [59]. Serum levels of VEGF increased significantly immediately after an average of 17 min of physical exercise [54]. Acute physical exercise increased serum levels of VEGF immediately after and 2 h post-exercise [60]. More studies would be necessary to verify the effects of gardening on VEGF.

## 5. Conclusions

Participants in the present study exhibited significantly increased levels of the brain nerve growth factors BDNF and PDGF by performing 20-min gardening activities with low to moderate intensity. This study revealed the potential of a short-term gardening activity for memory improvement in senior individuals and provided scientific evidence of the therapeutic mechanisms of gardening for memory. Future studies need to measure the changes in brain nerve growth factors in a long-term gardening activity intervention to verify the effects on memory. A follow-up study would be interesting to determine the effect continuance time for brain nerve growth factors and memory by gardening intervention. Moreover, future study needs to verify the therapeutic mechanisms of gardening intervention for brain nerve growth factors, rather than only focusing on the effects of gardening intervention as a physical activity. It would be valuable to compare differences among age ranges, between the sexes, and among subjects with different types of diseases and/or disabilities.

## Figures and Tables

**Table 1:** Gardening activities, making an vegetable garden plot performed by the subjects in the study of benefits gardening activities for memory according to measurement of brain growth factor levels.

Activity	Mean Time (min)	Description	Estimated MET Values ^1^
Cleaning garden plot	2	Removing weeds and fallen leaves in the garden plot	3.4
Digging	5	Digging a 1m (w) × 1.8 m (l) garden plot with a shovel (1.3 kg)	4.5
Fertilizing	3	Spreading fertilizer using a bucket (22 cm (d) × 9 cm (h); average 8.1 L) on the garden plot and then mixing it into the soil using a shovel	4.0
Raking	3	Raking the garden plot using a hand rake (0.9 kg), then making four furrows using a hand rake (0.9 kg)	3.4
Transplanting plant	5	Transplanting lettuce plants (average 23 plants) into the garden plot using a hand trowel (0.1 kg)	2.9
Watering using a watering can	2	Watering plants using a watering can (average 5.7 L)	2.8
(Total 20 min)		(Mean 3.5 METs)	

^1^ MET = metabolic equivalents.

**Table 2:** Demographic characteristics of the participants (*n* = 41).

Variable	Gardening Intervention Group
Sex	
Male	13 (31.7) ^1^
Female	28 (68.3)
Age (years)	76.6 ± 6.0 ^1^
Height (cm)	154.7 ± 8.1
Body composition	
Body weight (kg)	60.3 ± 10.1
Body mass index (kg/m^2^)	25.4 ± 3.8
Lean mass (kg)	37.6 ± 6.1
Fat mass (kg)	19.6 ± 7.2
Percent fat (%)	31.5 ± 8.7
Age-adjusted maximum heart rate (beats/min)	143.4 ± 6.0
Current disease	
Diabetes	7 (28.0)
Hyperlipidemia	5 (20.0)
Musculoskeletal	4 (16.0)
Genito-urinary	2 (8.0)
Respiratory	2 (8.0)
Gastrointestinal	2 (8.0)
Thyroid	2 (8.0)
Cerebral infarction	1 (4.0)
Current medications	
Blood pressure	24 (52.2)
Diabetes mellitus	7 (15.2)
Cholesterol	6 (13.0)
Antiarthritic	4 (8.7)
Thyroid	2 (4.4)
Gastrointestinal	2 (4.4)
Prostate	1 (2.2)

^1^ Data presented as mean ± standard deviation or *n* (%).

**Table 3:** The effects of a 20-min gardening activity program on brain nerve growth factor levels in senior individuals (*n* = 41).

Measurement ^1^	BDNF (ng/mL)	PDGF (pg/mL)	VEGF (pg/mL)
	**Mean ± SD**		
Pre-intervention	53.75 ± 21.49	3477.46 ± 1171.82	338.69 ± 171.64
Post-intervention	58.26 ± 23.40	3945.80 ± 1372.26	325.83 ± 145.98
*p* ^2^	0.038 *	0.001 **	0.126 NS

^1^ BDNF = brain-derived neurotrophic factor; PDGF = platelet-derived growth factor; VEGF = vascular endothelial growth factor. ^2^ NS, *, ** nonsignificant or significant at *p* < 0.05 or < 0.01, respectively.

**Table 4:** The effects of a 20-min gardening activity program on brain nerve factor levels of senior individuals by gender.

Variable	Male (*n* = 13)	Female (*n* = 28)	*p* ^1^
	Mean ± SD		
BDNF (ng/ml)			
Pre-intervention	58.7 ± 20.0	51.5 ± 22.1	0.362 NS
Post-intervention	67.2 ± 26.1	54.3 ± 21.5	0.132 NS
PDGF (pg/mL)			
Pre-intervention	3531.2 ± 1052.7	3450.6 ± 1252.5	0.863 NS
Post-intervention	4464.5 ± 1318.8	3686.4 ± 1355.7	0.146 NS
VEGF (pg/mL)			
Pre-intervention	375.2 ± 258.4	322.6 ± 119.4	0.405 NS
Post-intervention	364.5 ± 207.0	308.8 ± 110.6	0.298 NS

^1^ Independent samples *t*-test was used to compare means at *p* < 0.05. NS, Nonsignificant at *p* < 0.05.
